# Confirmation of covalently-linked structure and cell-death inducing activity in site-specific chemical conjugates of human Fas ligand extracellular domain

**DOI:** 10.1186/s13104-018-3501-8

**Published:** 2018-06-15

**Authors:** Michiro Muraki, Kiyonori Hirota

**Affiliations:** Biomedical Research Institute, National Institute of Advanced Industrial Science and Technology (AIST), Central 6, 1-1-1 Higashi, Tsukuba, Ibaraki 305-8566 Japan

**Keywords:** Human Fas ligand, Extracellular domain, Site-specific, Chemical conjugate, Covalent link, Cell-death inducing activity, MALDI-TOF mass-spectrometry, MTT assay

## Abstract

**Objective:**

In this study, we aimed to identify the structural components and to clarify the biological activity in the site-specific conjugates of human Fas ligand extracellular domain (hFasLECD) with either fluorescein moiety (FL) or chicken egg-white avidin (Avi). The conjugates were characterized by molecular-weight measurement using MALDI-TOF mass-spectrometric analysis and by cell-death inducing activity measurement against a human colorectal cancer cell line, HT-29, using MTT cell-viability assay. Pretreatment effect with human interferon-γ (IFN-γ) on the cell-death inducing activity was evaluated.

**Results:**

The mass-spectrometric analysis of the hFasLECD-Avi conjugate showed that it was possible to detect the signal peak of molecular-weight to electric charge (m/z) derived from the component involved in the covalent linking as the sum of the molecular-weight of unconjugated hFasLECD- and Avi-derivative subunits, in addition to the signals from each corresponding subunit component irrelevant to the covalent linking. The cell-viability assay revealed that both conjugates possessed a remarkable death-inducing activity against HT-29 cells in synergy with the pretreatment using human IFN-γ. Following 24 h pretreatment with 100 IU/ml of human IFN-γ, almost no viable cells existed after 72 h treatment with either 100 or 1000 ng/ml of FL-hFasLECD and hFasLECD-Avi conjugates.

**Electronic supplementary material:**

The online version of this article (10.1186/s13104-018-3501-8) contains supplementary material, which is available to authorized users.

## Introduction

Human Fas ligand (hFasL) is produced in many immune cells, predominantly by cytotoxic T-lymphocytes (CTL) cells and natural killer (NK) cells, and plays a central role of a major cell-death inducing factor in the elimination of diseased cells such as cancer cells and virally-infected cells in the human body [1]. The extracellular domain part of hFasL (hFasLECD) forms a complex with trimeric Fas receptors on the cell surface to trigger apoptosis of the target cells, which makes this domain attractive as a potential component of novel cytotoxic molecular tools for medical applications [2]. The soluble hFasLECD (26 kDa), excised from whole hFasL molecule (40 kDa) by matrix metalloproteinase-7, exhibited a substantially weakened cell-injuring activity [3–5], which can protect tumor cells from death caused by chemotherapeutic drugs [6, 7].

In previous studies, we developed an efficient secretory expression system of hFasLECD in *Pichia pastoris* [2, 8]. The derivatives of truncated hFasLECD (aa 139–281), containing site-specific chemical modifications of the Cys residue introduced at the amino-terminally attached tag sequence, retained the specific binding activity toward human Fas receptor extracellular domain. One of the derivatives showed the cell-death inducing activity against a human colorectal cancer cell-line, HT-29 cells, after cross-linking by an antibody [9]. The production of site-specific chemical conjugates was possible either by direct modification of the Cys residue with maleimide compounds [10] or by cyclo-addition reaction between *trans*-cyclooctene group (TCO) and methyltetrazine group (MTZ) [11]. The latter reaction was applicable to the conjugation of not only low molecular-weight compounds but also other functional proteins with molecular-weight similar to hFasLECD, such as egg-white avidin (Avi) and rabbit F(ab′) fragment, under mild conditions.

In this study, in order to directly identify structural components in the hFasLECD-Avi conjugate, the conjugate was characterized by MALDI-TOF mass-spectrometric analysis. A proinflammatory cytokine, interferon-γ (IFN-γ), also secreted from innate and adaptive immune cells, including CTL cells and NK cells, can induce intracellular apoptotic death progression [12]. IFN-γ has been suggested to sensitize antiproliferative agents-mediated apoptosis against several types of cells, either by increasing the expression level or by causing the re-localization of Fas receptor on the surface of target cells [13–17]. To explore such potentiating effects of IFN-γ in the case of external treatment with our site-specific chemical conjugates of hFasLECD, the cell-death inducing activity of fluorescein moiety-conjugated hFasLECD (FL-hFasLECD) and hFasLECD-Avi conjugate against HT-29 cells was evaluated in the presence or absence of pretreatment with IFN-γ using MTT cell-viability assay.

## Main text

### Materials and methods

hFasLECD-Avi conjugate was newly prepared according to the procedures in the previous paper [11]. The sample contained less than 10% of the unconjugated proteins as judged by the profile of size-exclusion chromatography (Additional file 1). FL-hFasLECD was prepared as described previously [10]. The conjugation ratio of fluorescein moiety at the reactive Cys residues was estimated to be 83%. Fluorescein conjugated bovine serum albumin (FL-BSA) was purchased from Thermo Fisher Scientific (A23015) and used as the negative control sample. Animal-derived-free recombinant human IFN-γ for cell biology was obtained from Wako Pure Chem. (093-06111) and used as a stock solution (1.0 mg/ml) dissolved in pure water. The biological activity and the endotoxin level were estimated to be 10 IU/ng and less than 0.01 ng/μg, respectively. All samples were kept frozen at below 253 K until use in the experiments.

The MALDI-TOF mass-spectrometric analysis was conducted by Brucker Ultra flex III-MALDI TOF/TOF mass-spectrometer in linear mode under the positive ionization condition using sinapinic acid as the matrix. Whole molecular-weight to electric charge ratio (m/z) measurement range was 10,000–300,000. Multiple peaks derived from BSA (average m/z: 5+, 13,287.00; 4+, 16,608.50; 3+, 22,144.30; 2+, 33,215.50; 1+, 66,430.00) were used for the calibration as the external standards. The hFasLECD-Avi conjugate at the concentration of 710 μg/ml in 25 mM Tris hydrochloride plus 15 mM sodium chloride (pH 7.5) was threefold diluted with pure water and subjected to analysis after mixed with the matrix solution at 1:1 ratio. Under the same condition, the measurement using matrix solution alone was performed to obtain control blank data for judging whether the peaks from the sample were true signals or not.

Human colorectal adenocarcinoma cell line, HT-29 cells (catalog number: EC91072201-GO, ECACC), used for the cell-viability measurement, were purchased from DS-Pharma Biomedical. The cells were maintained in McCoy’s 5A (Modified) medium (Thermo Fisher, 08457-55) supplemented with 10% FBS (Thermo Fisher, 10437-028) and 2% Penicillin–Streptomycin mixed solution (Nacalai Tesque, 09367-34) at 310 K in a 95% humidified air—5% CO_2_ incubator. Cell passages were carried out at 80% confluence at a ratio of 1:3. HT-29 cells were seeded at 3 × 10^3^ cells/well in 150 μl of the medium in poly-l-lysine coated 96 wells microplates (Iwaki), and allowed to attach and grow for 24 h. Then, 50 μl of the medium containing IFN-γ (0, 0.1, 1.0, 10 or 100 IU/ml) was added and incubated for another 24 h. After that, the medium was changed to 200 μl of that containing either hFasLECD conjugate, FL-BSA or PBS alone sample, and further incubated for 4–72 h. Four hours earlier than the time of absorbance measurement, 10 μl of MTT solution (5 mg/ml in PBS) was added. The culture medium was removed, and 200 μl each of DMSO was added to dissolve the contents. Then, the absorbance at 535 nm was measured immediately. The serial dilution of the test samples was conducted using PBS. The cell-viability was evaluated as the value relative to the average of PBS alone sample. Four independent experimental data under each treatment condition were used for the calculation of average value and standard error of mean.

### Results

#### Identification of structural components in hFasLECD-Avi conjugate

**Fig. 1:**
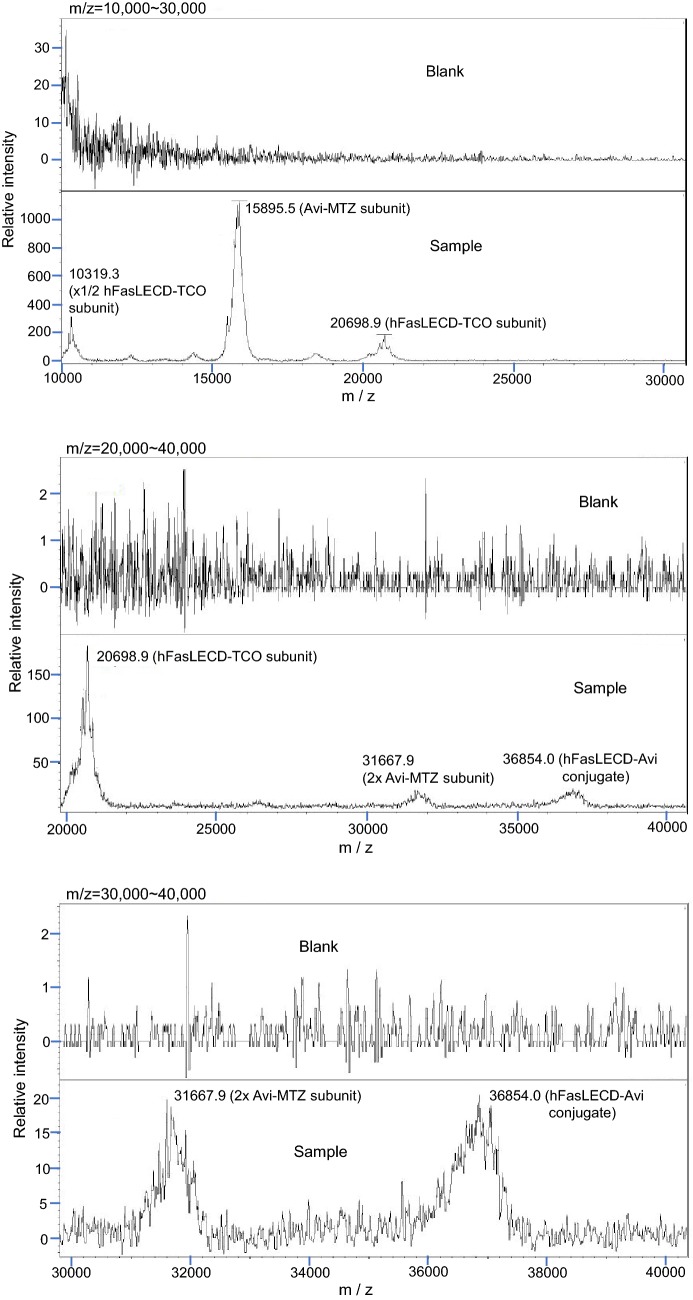
MALDI-TOF mass-spectrometric analysis of hFasLECD-Avi conjugate. Panels: upper, m/z = 10,000–30,000; middle, m/z = 20,000–40,000; lower, m/z = 30,000–40,000. Sample, hFasLECD-Avi conjugate mixed with matrix solution; blank, matrix solution alone. Representative peaks of the identified subunits were labeled with the m/z values and the names of possible components

The MALDI-TOF mass-spectrometric analysis identified both signals derived from the subunits involved in and that irrelevant to the covalent linking. Figure 1 shows the spectra in m/z ranges of 10,000–30,000, 20,000–40,000 and 30,000–40,000. The signal derived from the covalently-linked subunits in hFasLECD-Avi conjugate was detected as the m/z peak at 36,854.0 (middle and lower panels). While, the signals, which can be attributed to the unconjugated hFasLECD-TCO and Avi-MTZ subunits, were observed as the m/z peaks at 10,319.3, 20,698.9 (upper and middle panels) and 15,895.5, 31,667.9 (all panels), respectively. The peak m/z value of the covalently-linked subunits was detected as 99.3% total of the corresponding values (20,698.9 and 15,895.5) for both unconjugated protein-derivative subunits. No appreciable m/z peak was present above 40,000, suggesting the absence of multiply-conjugated molecules (Additional file 2). These results showed a good agreement with the data of the SDS-PAGE and the size-exclusion chromatography analysis performed previously [11]. Consequently, it was confirmed that the conjugated sample possessed a covalent bond between the subunits belonging to hFasLECD and Avi and was suggested to be mostly composed of the 1:1 adducts of each protein.

#### Evaluation of cell-death inducing activity of FL-hFasLECD and hFasLECD-Avi conjugates

**Fig. 2:**
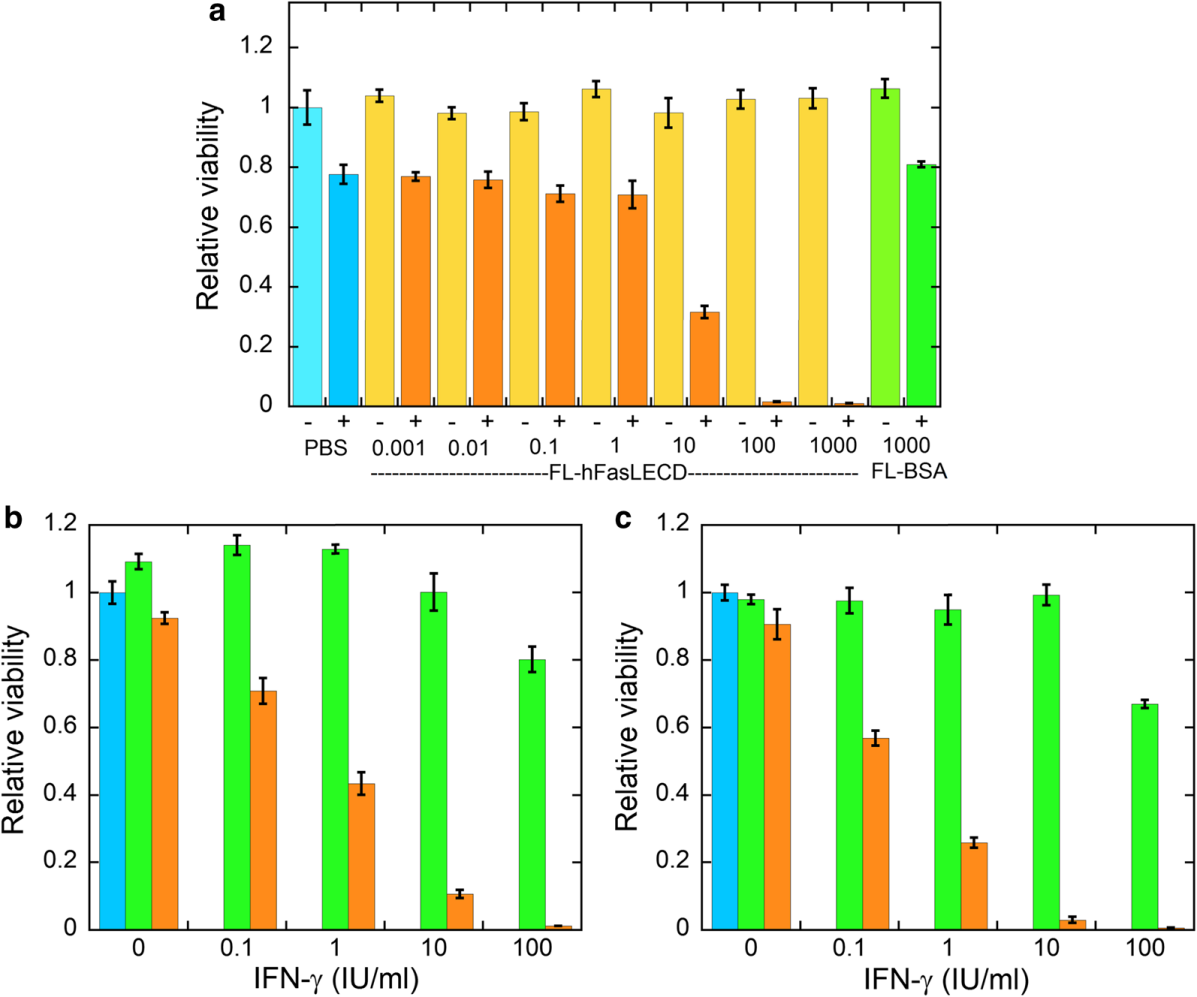
Cell-death inducing activity of FL-hFasLECD conjugate. **a** Concentration dependency of FL-hFasLECD. The numerals represent the treatment concentration of FL-hFasLECD and FL-BSA in ng/ml. PBS, negative control buffer used for the dilution of samples; pretreatment condition: −, no IFN-γ; +, 100 IU/ml of IFN-γ. Treatment time 72 h. **b**, **c** Concentration dependency of IFN-γ. Colors: blue, PBS alone; green, FL-BSA; orange, FL-hFasLECD. Sample concentration: **b** 100 ng/ml; **c** 1000 ng/ml. Treatment time 72 h. Standard error of mean under each experimental condition was included as an error bar

Cell-death inducing activity of FL-hFasLECD and the same purified hFasLECD-Avi conjugates were evaluated using MTT cell-viability assay. Figure 2 illustrates the concentration dependency of FL-hFasLECD on the relative viability of HT-29 cells in either the presence or absence of the pretreatment with human IFN-γ (100 IU/ml) (panel a). Also, the IFN-γ concentration dependency during the pretreatment prior to the addition of FL-hFasLECD or FL-BSA is shown (panels b and c). As shown in Fig. 2a, the relative viability of HT-29 cells was not essentially affected by the treatment with FL-hFasLECD under the pretreatment condition with no IFN-γ. The relative viability remained the same as in the cases of FL-BSA (1000 ng/ml) and PBS alone. In contrast, a prominent reduction in the cell-viability was observed for the treatment with FL-hFasLECD at concentrations of higher than 10 ng/ml after the IFN-γ pretreatment (100 IU/ml) for 24 h (panel a). It should be noted that also in the cases of FL-BSA and PBS alone, the pretreatment with 100 IU/ml of IFN-γ significantly reduced the cell-viability, which may reflect the small up-regulation of endogenous Fas ligand expression in HT-29 cells [13]. The IFN-γ concentration in the pretreatment affected the cell-viability (panels b and c). The cell-death induction against HT-29 cells was remarkably sensitized by the IFN-γ pretreatment concentration-dependently in the treatment with FL-hFasLECD at concentrations of both 100 and 1000 ng/ml. This stimulating effect was always substantial in the range from 0.1 to 100 IU/ml of IFN-γ in comparison with the cases of FL-BSA. Under the conditions using 100 IU/ml of IFN-γ and 100 or 1000 ng/ml of FL-hFasLECD, almost no viable cell was observed after 72 h treatment.

**Fig. 3:**
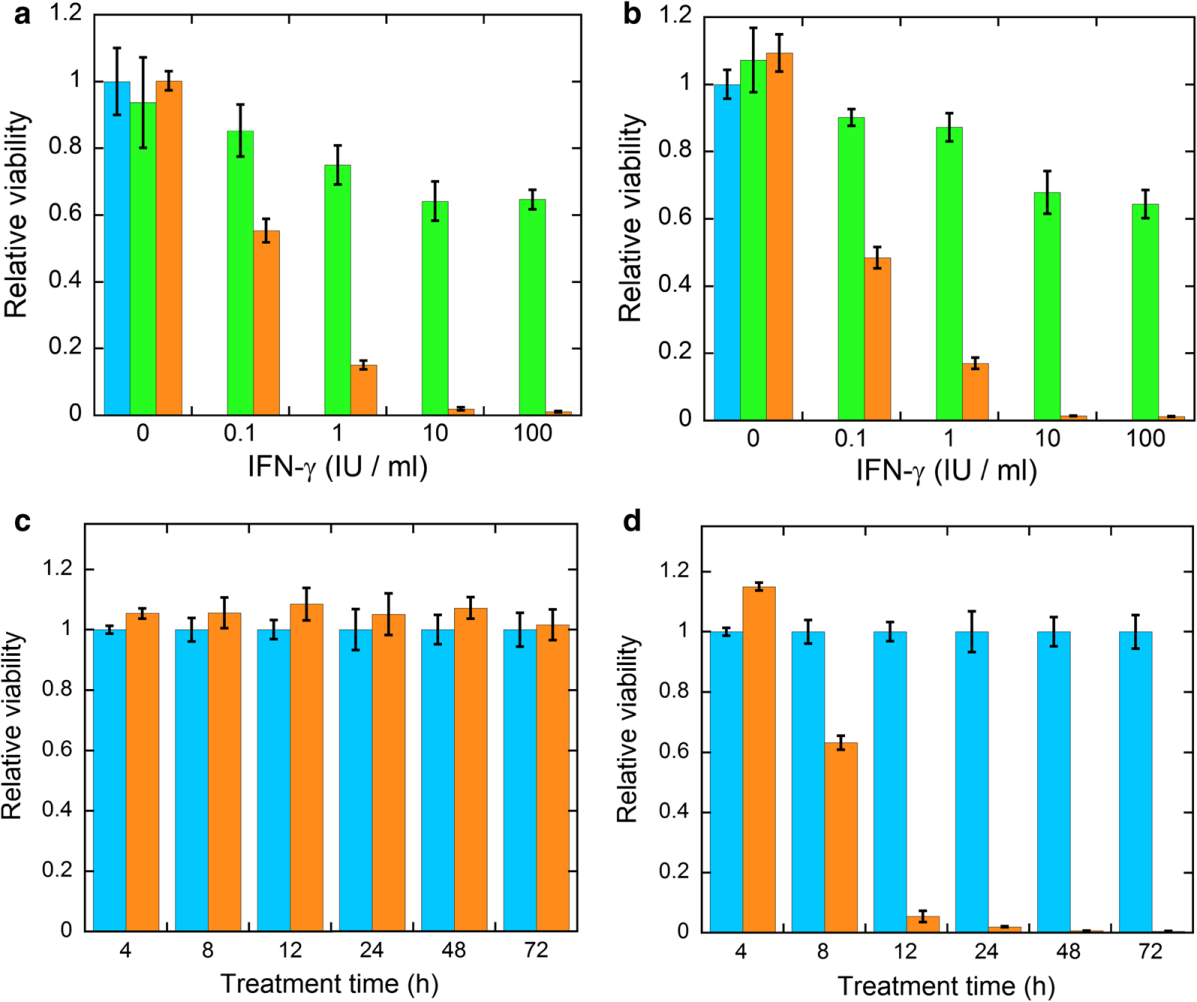
Cell-death inducing activity of hFasLECD-Avi conjugate. **a**, **b** Concentration dependency of IFN-γ. Colors: blue, PBS alone; green, FL-BSA; orange, hFasLECD-Avi. Sample concentration: **a** 100 ng/ml; **b** 1000 ng/ml. Treatment time, 72 h. **c**, **d** Treatment-time dependency. Colors: blue, PBS alone; orange, hFasLECD-Avi (100 ng/ml). Pretreatment condition: **c** no IFN-γ; **d** 100 IU/ml of IFN-γ. Standard error of mean under each experimental condition was included as an error bar

In Fig. 3, the experimental results of hFasLECD-Avi conjugate against HT-29 cells are summarized. The cell-viability after the treatment with either 100 or 1000 ng/ml of hFasLECD-Avi conjugate was again highly reduced by the pretreatment with 0.1–100 IU/ml of IFN-γ. The sensitizing effect was IFN-γ concentration dependent as was the case of FL-hFasLECD (panels a and b). Following 24 h pretreatment with 10 or 100 IU/ml of IFN-γ, almost no cells remained alive after 72 h treatment with either 100 or 1000 ng/ml of hFasLECD-Avi conjugate. For the purpose of investigating the initiation time of the cell-death, the cell-viability was checked after 4–72 h in the treatment with 100 ng/ml of hFasLECD-Avi conjugate, preceded by the pretreatment with either 0 or 100 IU/ml of IFN-γ (panels c and d). As expected, no significant difference in the cell-viability was observed between hFasLECD-Avi (100 ng/ml) and PBS alone samples in the case that IFN-γ was not included in the pretreatment. In contrast, a significant reduction in the cell-viability started from 8 h, and the level of cell-death was almost maximized after 12–24 h in the case that 100 IU/ml of IFN-γ was included. In both cases of FL-hFasLECD and hFasLECD-Avi, the microscopic observation revealed that the morphology of HT-29 cells changed from a transparent, expanded form to a dense, shrunk shape, which indicated the apoptotic cell-death (Additional file 3).

### Discussion

In the MALDI-TOF mass-spectrometric analysis of hFasLECD-Avi conjugate, the intensity of the m/z peak derived from the component composed of covalently-linked hFasLECD-TCO and Avi-MTZ subunits appeared considerably weak relative to that from each single subunit derivative alone (Fig. 1), even after taking relative abundance of each component in the 1:1 conjugate into account. This suggested the difficulty in ionization of the linked component. The generally broadened peaks could be ascribed to the heterogeneity of the carbohydrate chains attached to the hFasLECD-TCO and Avi-MTZ subunits.

A remarkable cell-death of a colorectal cancer cell-line, HT-29 cells, was triggered by both site-specific conjugates of hFasLECD in synergy with the human IFN-γ pretreatment. This can be considered to be a mimic of the IFN-γ release by CTL and NK cells toward malignant cells in the human body. Recently, it was demonstrated that the treatment with IFN-γ augmented proliferation inhibition or apoptosis execution against various types of cancer cells by activating several signal transduction systems, including JAK-STAT 1 pathway [18, 19]. The enhancement of cell-death inducing activity in this study is also supposed to be linked with such signaling mechanisms.

### Conclusions

In conclusion, the above experimental results confirmed the covalently-linked structure and the cell-death inducing activity in the site-specific chemical conjugates of hFasLECD. It is important to note that the death of HT-29 cells was strongly induced with the synergistic assistance of IFN-γ pretreatment under the conditions devoid of any cross-linking antibody. Our present study reinforces the way to the development of novel, clinically valuable molecular tools using hFasLECD as the conjugational component with the concrete structural and functional evidences. The combination with useful targeting molecules to diseased cells in the conjugation would contribute to the future development of the molecular tools aimed at medical applications.

### Limitations

The major limitations of this study are that only two site-specific chemical conjugates of hFasLECD and a single cancer cell-line were used as the test samples for the examinations and the cells employed in the cell-viability assay, respectively. There remains a necessity for the evaluation using various types of conjugate samples and target cell-lines in order to generalize the conclusions.

## Additional files

**Additional file 1.** Size-exclusion chromatography profile of the hFasLECD-Avi conjugate sample. 40 μg of the sample was resolved using a Superdex 200 Increase 10/300 GL column (GE healthcare) under the conditions of 50 mM Tris–HCl plus 150 mM NaCl (pH 7.5) as the elution buffer and flow rate of 0.75 ml/min. Absorbance at 280 nm was used for the peak detection.

**Additional file 2.** MALDI-TOF mass-spectrometric analysis of hFasLECD-Avi conjugate in the m/z measurement range between 10,000 and 300,000. Representative peaks of the identified subunits were labeled with the m/z values and the names of possible components. The measurement conditions are described in the text.

**Additional file 3.** Effect on cell morphology of HT-29 cells. After 72 h treatment with 100 ng/ml of hFasLECD conjugates. Panels: a, FL-hFasLECD, after 24 h pretreatment with PBS buffer alone (left) and 100 IU/ml of IFN-γ (right). Scale bar: 100 μm; b, hFasLECD-Avi, after 24 h pretreatment with PBS buffer alone (left) and 100 IU/ml of IFN-γ (right).

## Abbreviations

aa: amino-acid residue number
Avi: chicken egg-white avidin
BSA: bovine serum albumin
Cys: l-cysteine
DMSO: dimethyl sulfoxide
FL: fluorescein moiety
hFasLECD: human Fas ligand extracellular domain
IFN-γ: interferon gamma
MALDI-TOF: matrix assisted laser desorption ionization-time of flight
MTT: 3-(4,5-dimethylthiazol-2-yl 2,5-diphenyl-tetrazolium bromide
MTZ: 6-methyltetrazine group
m/z: molecular-weight to electric charge ratio
PBS: phosphate-buffered saline
TCO: *trans*-cyclooctene group
