# Supplementary material for: Confirmation of covalently-linked structure and cell-death inducing activity in site-specific chemical conjugates of human Fas ligand extracellular domain
Source: BMC Res Notes. 2018 Jun 15;11:395. doi: 10.1186/s13104-018-3501-8 (PMC6003068; doi:10.1186/s13104-018-3501-8)
Supplement: Supplementary file 1 — Additional file 1. Size-exclusion chromatography profile of the hFasLECD-Avi conjugate sample. 40 μg of the sample was resolved using a Superdex 200 Increase 10/300 GL column (GE healthcare) under the conditions of 50 mM Tris–HCl plus 150 mM NaCl (pH 7.5) as the elution buffer and flow rate of 0.75 ml/min. Absorbance at 280 nm was used for the peak detection. [file 13104_2018_3501_MOESM1_ESM.pptx]

## Slide 1
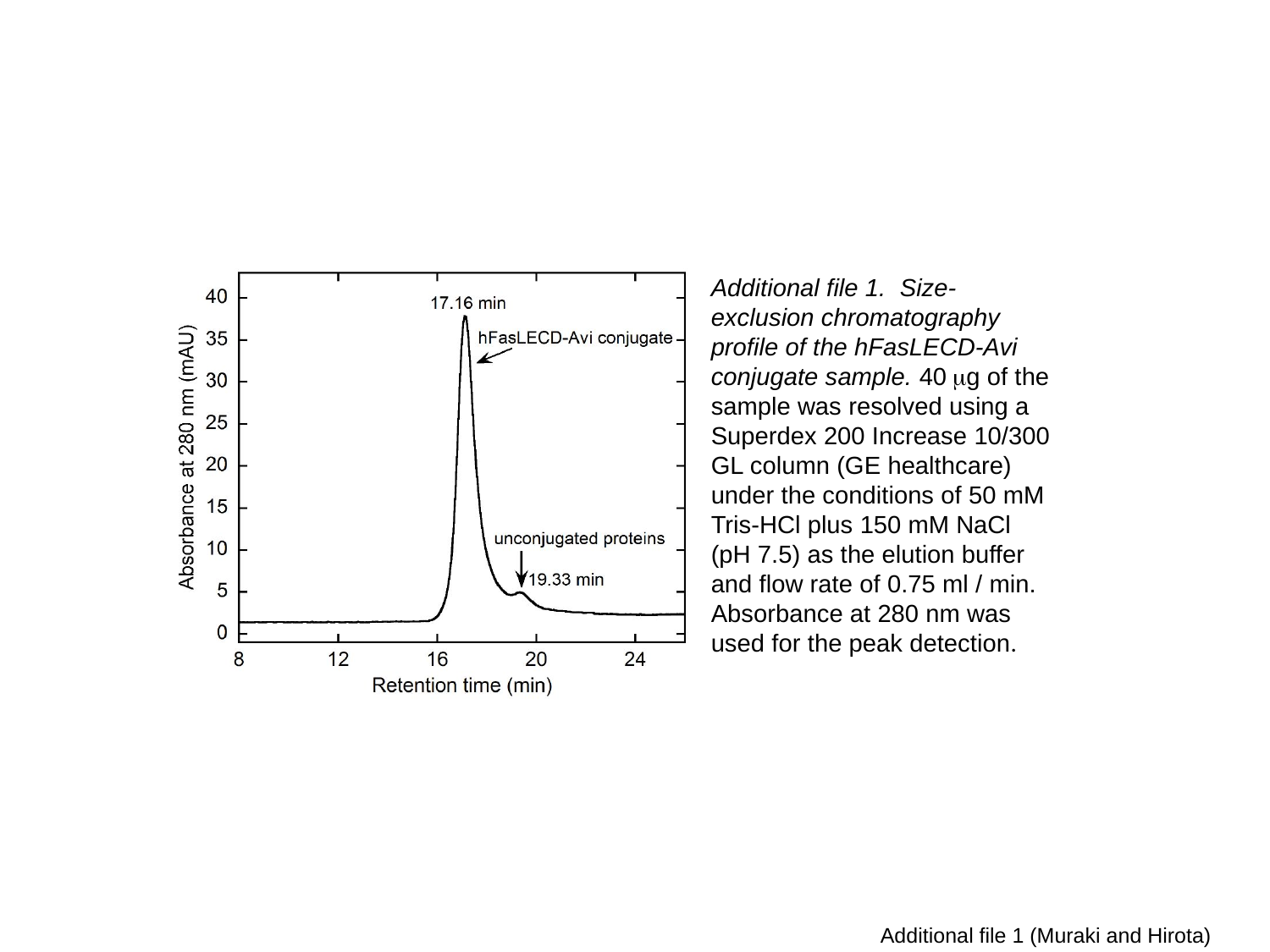

Additional file 1. Size-exclusion chromatography profile of the hFasLECD-Avi conjugate sample. 40 mg of the sample was resolved using a Superdex 200 Increase 10/300 GL column (GE healthcare) under the conditions of 50 mM Tris-HCl plus 150 mM NaCl (pH 7.5) as the elution buffer and flow rate of 0.75 ml / min. Absorbance at 280 nm was used for the peak detection.
Additional file 1 (Muraki and Hirota)
