# Supplementary material for: Confirmation of covalently-linked structure and cell-death inducing activity in site-specific chemical conjugates of human Fas ligand extracellular domain
Source: BMC Res Notes. 2018 Jun 15;11:395. doi: 10.1186/s13104-018-3501-8 (PMC6003068; doi:10.1186/s13104-018-3501-8)
Supplement: Supplementary file 2 — Additional file 2. MALDI-TOF mass-spectrometric analysis of hFasLECD-Avi conjugate in the m/z measurement range between 10,000 and 300,000. Representative peaks of the identified subunits were labeled with the m/z values and the names of possible components. The measurement conditions are described in the text. [file 13104_2018_3501_MOESM2_ESM.pptx]

## Slide 1
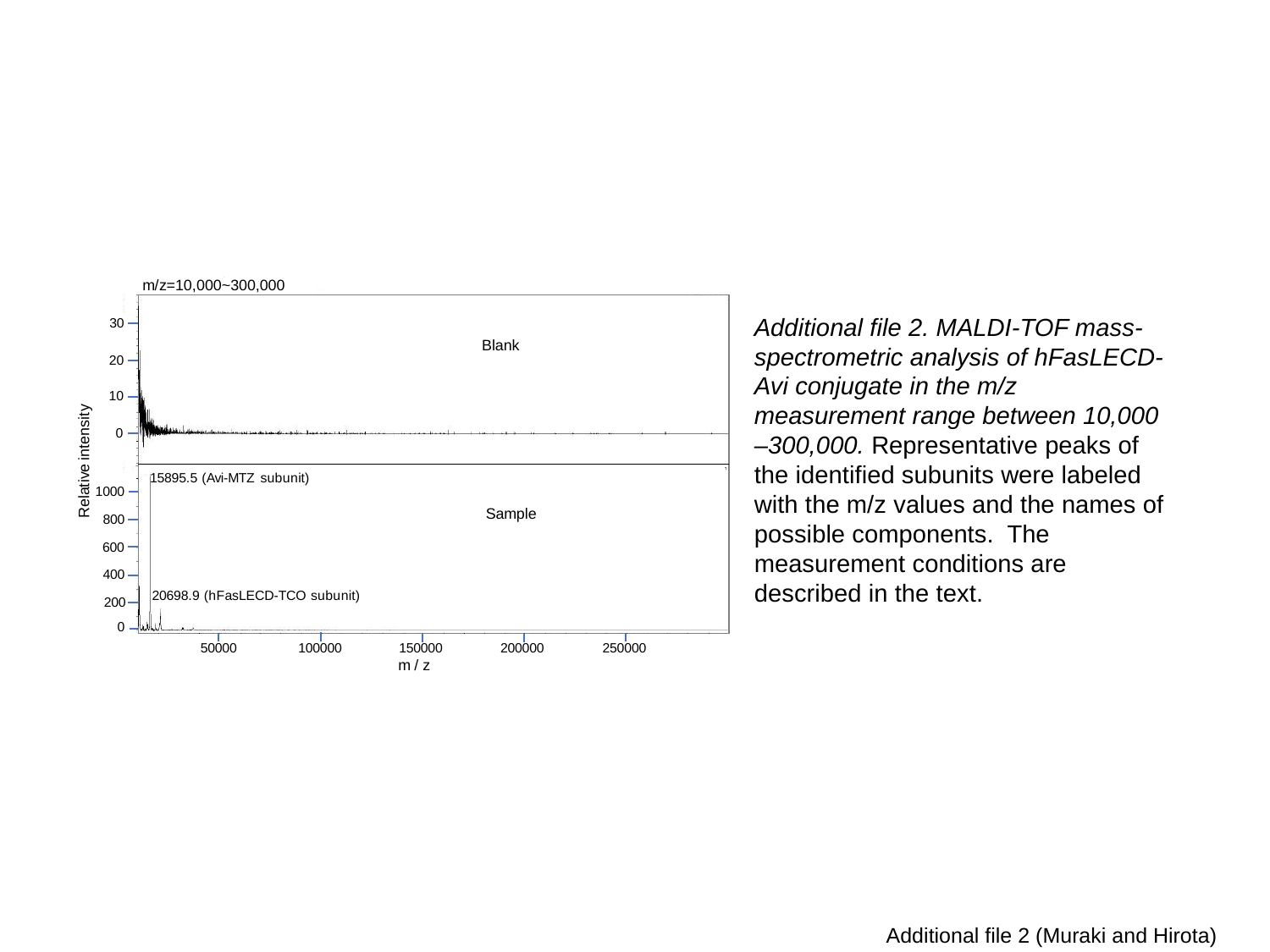

Additional file 2. MALDI-TOF mass-spectrometric analysis of hFasLECD-Avi conjugate in the m/z measurement range between 10,000 –300,000. Representative peaks of the identified subunits were labeled with the m/z values and the names of possible components. The measurement conditions are described in the text.
Additional file 2 (Muraki and Hirota)
