# Supplementary material for: Confirmation of covalently-linked structure and cell-death inducing activity in site-specific chemical conjugates of human Fas ligand extracellular domain
Source: BMC Res Notes. 2018 Jun 15;11:395. doi: 10.1186/s13104-018-3501-8 (PMC6003068; doi:10.1186/s13104-018-3501-8)
Supplement: Supplementary file 3 — Additional file 3. Effect on cell morphology of HT-29 cells. After 72 h treatment with 100 ng/ml of hFasLECD conjugates. Panels: a, FL-hFasLECD, after 24 h pretreatment with PBS buffer alone (left) and 100 IU/ml of IFN-γ (right). Scale bar: 100 μm; b, hFasLECD-Avi, after 24 h pretreatment with PBS buffer alone (left) and 100 IU/ml of IFN-γ (right). [file 13104_2018_3501_MOESM3_ESM.pptx]

## Slide 1
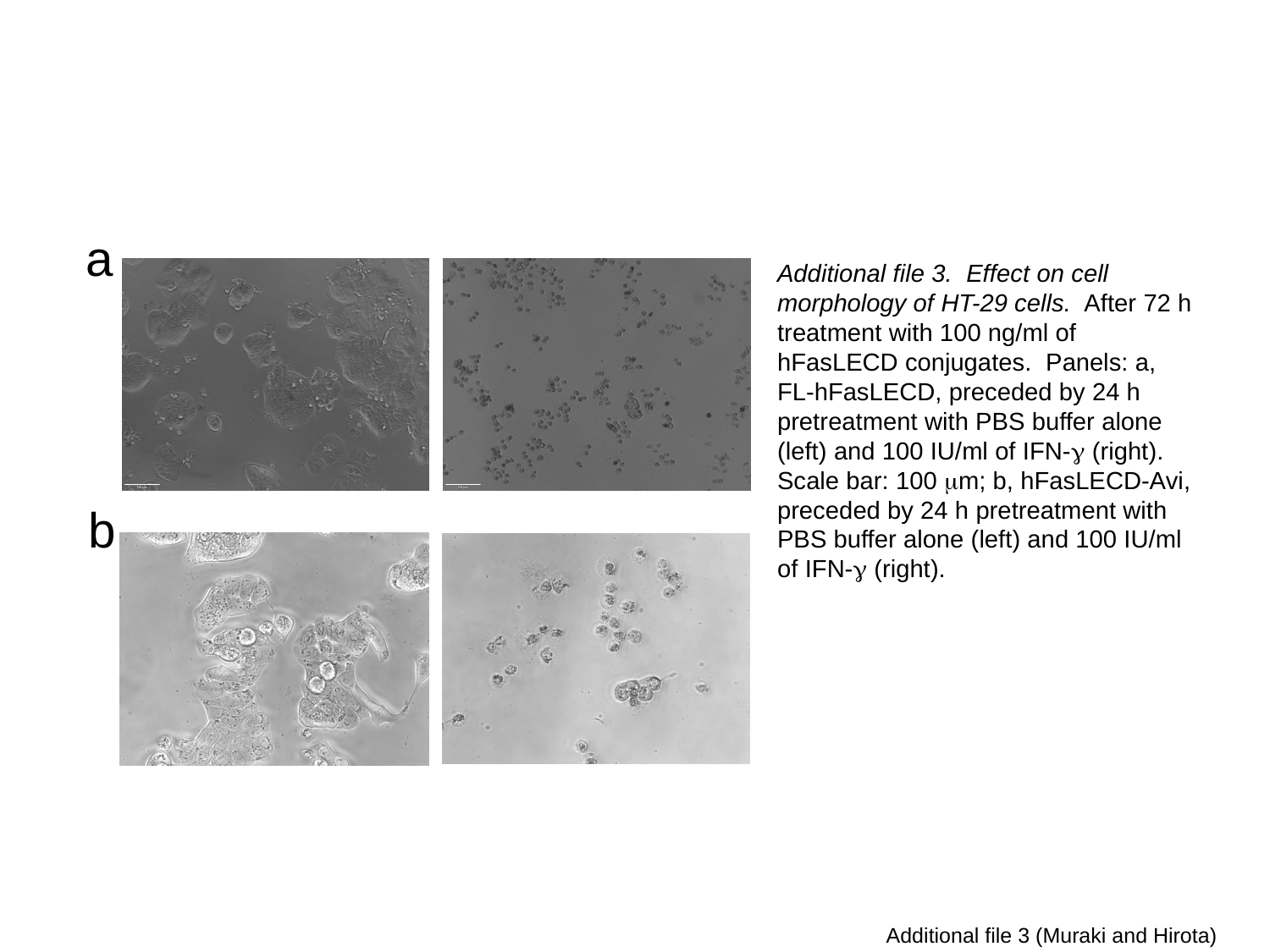

a
b
Additional file 3. Effect on cell morphology of HT-29 cells. After 72 h treatment with 100 ng/ml of hFasLECD conjugates. Panels: a, FL-hFasLECD, preceded by 24 h pretreatment with PBS buffer alone (left) and 100 IU/ml of IFN-g (right). Scale bar: 100 mm; b, hFasLECD-Avi, preceded by 24 h pretreatment with PBS buffer alone (left) and 100 IU/ml of IFN-g (right).
Additional file 3 (Muraki and Hirota)
